# Simulation Study on Direct Contact Membrane Distillation Modules for High-Concentration NaCl Solution

**DOI:** 10.3390/membranes10080179

**Published:** 2020-08-05

**Authors:** Weiming Ni, Yongli Li, Juezhen Zhao, Gaoyuan Zhang, Xiaoze Du, Yingchao Dong

**Affiliations:** 1 Key Laboratory of Power Station Energy Transfer Conversion and System (North China Electric Power University), Ministry of Education, Beijing 102206, China; niweiming@ncepu.edu.cn (W.N.); zjz_jinhuai@126.com (J.Z.); gaoyuan@ncepu.edu.cn (G.Z.); 2 School of Energy and Power Engineering, Lanzhou University of Technology, Lanzhou 730050, China; 3 School of Environmental Science and Technology, Dalian University of Technology, Dalian 116024, China; ycdong@dlut.edu.cn

**Keywords:** direct contact membrane distillation, evaporation efficiency, optimal membrane thickness, high-salt wastewater

## Abstract

Membrane distillation technology, as a new membrane-based water treatment technology that combines the membrane technology and evaporation process, has the advantages of using low-grade heat, working at atmospheric pressure with simple configuration, etc. In this study, heat and mass transfer were coupled at the membrane surfaces through the user-defined function program. The effects of feed temperature, feed velocity and permeate velocity on temperature polarization were mainly investigated for a high-concentration NaCl solution. The temperature polarization was increased with the increase of feed temperature and the decrease of feed and permeate velocity. The effects of temperature, inlet velocity and solution concentration on the evaporation efficiency of the membrane module for co- and counter-current operations were investigated in detail. The counter-current operation performed better than co-current operation in most cases, except for the condition where the NaCl concentration was relatively low or the module length was long enough. In addition, the optimal membrane thickness for both PVDF and PTFE was studied. The optimal membrane thickness was found in the range of 10 to 20 μm, which corresponded to the highest permeate flux for the selected materials, pore size distribution, and operation conditions. Membrane material with lower thermal conductivity and larger porosity was prone to get higher permeate flux and had larger optimal membrane thickness. Increasing feed velocity or feed temperature could decrease the optimal membrane thickness.

## 1. Introduction

High-salt wastewater treatment attracts the attention of researchers and engineers in the field of wastewater treatment for production processes in chemical industries, seawater desalination, water recovery from desulfurization process in power plants, etc. In power plants, a large amount of desulfurization wastewater is produced in the process of flue gas desulfurization (FGD) and, in many countries, FGD wastewater is required to be recovered and reused due to environmental requirements and economic considerations. The process is usually composed of a pretreatment unit, after which wastewater contains mainly salt, and evaporation unit, where most of water is recovered accompanied even by a crystallization process. In the evaporation step, evaporation techniques such as mechanical vapor recompression (MVR) or membrane distillation (MD) are used for water recovery. MVR is popularly applied but consumes high power and leads to high cost. However, MD can make use of heat energy instead of power energy. In power plants, sufficient low-grade heat sources are available, which are not efficiently reused currently. From the point view of energy saving, one feasible method is to concentrate the solution close to saturation by MD, and then to further vaporize by MVR to separate completely water from salt. MD is a promising technology for desalting wastewater. It is a thermally driven separation process using a hydrophobic microporous membrane, different from the pressure-driven membrane process [1,2,3,4,5,6,7]. Heat and mass transfer through the membrane are both involved in the process [8]. The polarization effects due to concentration profile, or temperature profile, can lead to low vapor pressure difference, causing a performance reduction. Low-pressure operation condition and high rejection factors of non-volatile solutes, utilization of low-grade waste heat is the main advantage of the MD process [9]. Direct contact membrane distillation (DCMD) is a MD configuration where both feed and permeate solutions are in direct contact with the membrane. Vapor is condensed in the permeate side by direct contact with cooling water. DCMD is a simple configuration and has been widely studied [10].

DCMD performance is often characterized by permeate flux, temperature polarization, concentration polarization [11], and energy efficiency [12,13], etc. Temperature polarization and concentration polarization refer to the temperature or concentration difference between the bulk solution and the solution near the membrane interface, which results in a reduction of the driving force. Temperature polarization is caused by the existence of thermal gradient near the membrane surface. Concentration polarization is caused by the accumulation of solutes adjacent to the feed side of the membrane. It reduces the transmembrane vapor pressure difference [14]. There are several factors affecting the performance of DCMD, including the operation conditions, membrane characteristics, and flow patterns. Numerous studies have successfully investigated the heat and mass transfer mechanisms either by the using Nusselt and Sherwood numbers [8,15,16,17,18], or by computational fluid dynamics (CFD) simulation [19,20,21,22,23,24,25,26,27,28,29,30,31]. Unidimensional numbers based on specific parameters or physical assumptions offer a simplified method. It takes much less computational time, but cannot capture the local variation of polarization, permeate flux, and conductive heat loss along the membrane. CFD has the advantage of studying the full temperature, velocity, and concentration fields along the membrane. Some studies focused on the coupled simulation of momentum, heat and mass transfer [8,20], some were targeted at understanding the flow patterns on MD performance related to module orientation and design [19,24,28,30], and some investigated the influence of membrane properties, including membrane materials, thickness, porosity, and tortuosity [32,33,34,35,36]. It is generally accepted that an optimal membrane thickness exists due to the balance between mass transport and heat transfer [35] for membrane structures and the permeability can be improved by reducing tortuosity or by increasing porosity and pore size [32,33,34].

The CFD simulation of DCMD modules was employed to investigate the heat transfer and mass transfer of DCMD modules for concentrated NaCl solution. The evaporation process at the high-concentration state is more critical as it is close to the final step of MD process, and it was not well studied. In this work, the treatment of high salt solutions with MD was studied. The real conditions for treating desulfurization wastewater in power plants were used as boundary conditions to explore the influence of the temperatures of both permeate and feed solutions, as well as the flow rate of the feed solution. The salt concentration used in the current study was high, thus the concentration polarization could be negligible [37]. The effects of *T*_f_, *v*_f_, *v*_p_ and *c*_f_ on evaporation efficiency (EE) for co- and counter-current operations were investigated in detail. The optimal membrane thickness for various membranes was analyzed in different operation conditions. The performance of membrane distillation in high-salt solution including evaporation efficiency and permeate flux were evaluated for different flow patterns, membrane materials and operation conditions, which could provide guidance for optimizing DCMD processes.

## 2. Theoretical Background

**Figure 1:**
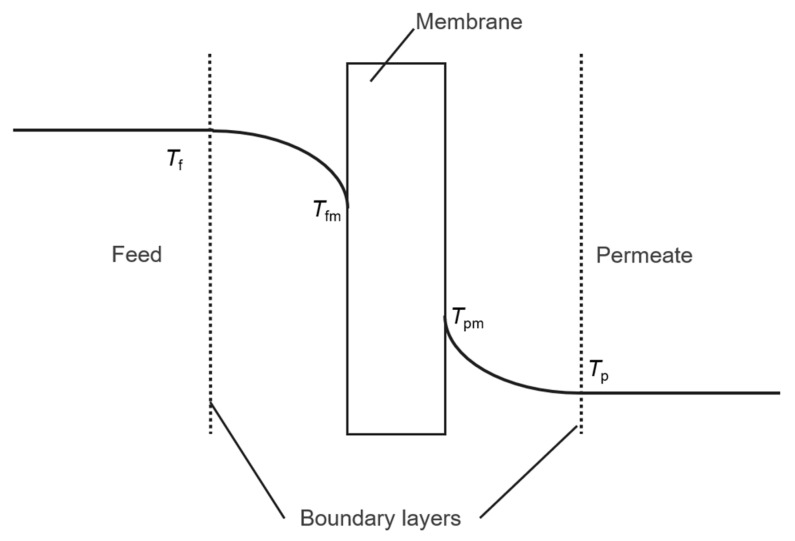
Heat transfer schematic diagram of direct contact membrane distillation (DCMD).

DCMD is the MD configuration in which both feed and permeate solutions are in direct contact with the hydrophobic porous membrane. The transmembrane temperature gradient serves as the driving force for mass transfer. As shown in Figure 1, heat was transferred from the feed solution to the heat boundary layer, and then the membrane surface, where water was vaporized. The heat loss from the feed solution was composed of the latent heat of vaporization, and the heat conduction of the membrane itself. The latter reduced the temperature difference between the two solutions. After, the vapor was diffused to the permeate side through the membrane. Vapor condensed at the interface. Conductive heat was transferred from the feed side to the permeate side of the membrane and then through the heat boundary layer.

The temperature polarization was schematically shown in Figure 1. Temperatures, *T*_fm_, *T*_pm_ at the membrane–liquid interfaces were different from bulk solution temperature, *T*_f_, *T*_p_, caused by the resistance to the heat flux across the membrane boundary [26]. Furthermore, a concentration boundary layer was also formed near the membrane surface of the liquid layer, so that the membrane surface solute concentration was higher than the bulk solute concentration, i.e., the concentration polarization [18]. Temperature and concentration polarization reduced the mass transfer driving force, leading to a reduction in permeation flux, which needed to be considered in the simulation.

The heat transfer from feed solution to membrane surface of feed side was given by,

(1) $$Q_{f}=h_{f}\left(T_{f}-T_{\mathrm{fm}}\right).$$

The heat transfer across the membrane consists of two parts: the latent heat of vaporization and the heat conduction. The latter one was considered as energy loss. The evaporation efficiency (EE) was defined as the ratio of the efficient heat due to vaporization and the total heat transfer through the membrane and was calculated as,

(2) $$EE=\frac{Q_{N}}{Q_{N}+Q_{c}},$$

where *Q_N_* and *Q*_c_ could be calculated as,

(3) $$Q_{N}=N\Delta H$$

(4) $$Q_{c}=\frac{k_{m}}{\delta }\left(T_{\mathrm{fm}}-T_{\mathrm{pm}}\right)$$

The heat transfer from the membrane surface of permeate side to permeate solution was given by,

(5) $$Q_{p}=h_{p}\left(T_{p}-T_{\mathrm{pm}}\right).$$

According to the conservation of energy,

(6) $$Q_{f}=Q_{N}+Q_{c}=Q_{p}.$$

The latent heat of vaporization was obtained by [8],

(7) $$\Delta H=-0.001351T_{\mathrm{fm}}^{2}-1.4461T_{\mathrm{fm}}+2986.5.$$

The thermal conductivity was obtained by [18],

(8) $$k_{m}=\varepsilon k_{g}+\left(1-\varepsilon \right)k_{s}.$$

When *T*_f_ ranged from 45 to 60 °C, the mean free path in DCMD was from 0.107 to 0.11 μm. The pore size of membrane ranged from 0.2 to 1.0 μm. Kn number ranged from 0.107 to 0.55. The mass transport took places via the combination of Knudsen diffusion and Molecular diffusion [38], and the mass transfer model was given by,

(9) $$N=\frac{\left(\frac{\varepsilon }{\lambda }\right)P_{T}D_{w\text{-}a}}{\delta RT_{m}}\times \ln\left(\frac{D_{\mathrm{Kn}}\left(P_{T}-P_{\mathrm{pm}}\right)+\left(\frac{\varepsilon }{\lambda }\right)P_{T}D_{w\text{-}a}}{D_{\mathrm{Kn}}\left(P_{T}-P_{\mathrm{fm}}\right)+\left(\frac{\varepsilon }{\lambda }\right)P_{T}D_{w\text{-}a}}\right)$$

(10) $$D_{\mathrm{kn}}=\frac{4}{3}\frac{\varepsilon d_{m}}{\lambda }\sqrt{\frac{RT_{m}}{2\pi m_{w}}}$$

(11) $$P_{\mathrm{fm}}=\left(1-x_{s}\right)P(T_{fm})\gamma_{w}.$$

The activity coefficient of water under different concentration conditions could be obtained by the following empirical formula [39]:

(12) $$\gamma_{w}=1-0.5x_{s}-10x_{s}^{2},$$

where *x*_s_ was the molar fraction of solute in the solution. *D*_w−a_ was evaluated by the empirical formula, given by [40],

(13) $$P_{T}D_{w\text{-}a}=\left(1.895\times 10^{-5}\right)T^{2.072}.$$

The water vapor pressure *P*_fm_, and *P*_pm_ were calculated through *T*_fm_, *T*_pm_ by Antoine equation,

(14) $$P\left(T\right)=\exp\left(23.1964-\frac{3816.44}{T-46.13}\right).$$

In the MD process, the temperature polarization was measured with the temperature polarization coefficient (TPC),

(15) $$TPC=\frac{T_{\mathrm{fm}}-T_{\mathrm{pm}}}{T_{f}-T_{p}}.$$

The concentration polarization was characterized using a concentration polarization coefficient (CPC), which was defined as the ratio of the membrane surface concentration to the feed inlet concentration,

(16) $$CPC=\frac{c_{\mathrm{fm}}}{c_{f}}.$$

## 3. Methodology

### 3.1. Geometry and Governing Equations

**Figure 2:**
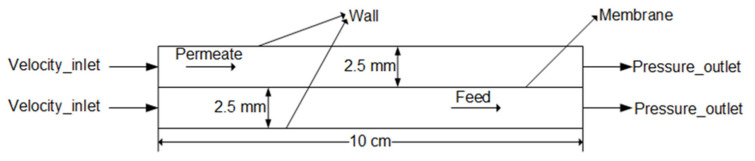
Schematics of the 2-D geometry model of the membrane module.

As shown in Figure 2, the geometry model was a rectangle flow channel which was 100 mm long, 2.5 mm high. The computational domain consisted of permeate and feed channels. Boundary conditions were set as shown in Figure 2. The inlet of permeate and feed channel were set to Velocity-Inlet. The outlet of permeate and feed channel were set to Pressure-Outlet. The membrane materials used in the simulation were shown in Table 1. The membrane module was placed horizontally. The NaCl solution was taken as feed solution, and pure water was used as the cooling substance. *T*_f_ was set from 45 to 75 °C according to the fact that the desulfurization wastewater temperature was about 50 °C [41]. The temperature difference of feed and permeate solution was from 20 to 50 °C. The flow rate was set from 0.05 to 0.25 m/s. In Section 4.3, in order to investigate the temperature field and EE of co- and counter-current operations obviously, the calculation domain of x direction was extended by three times.

In the current work, quadrilateral structured mesh was generated. In order to describe the fluid properties of the boundary layer near the membrane accurately, finer grids on both sides of the membrane were taken. The first layer of mesh on both sides of the film had a thickness of 10 μm and a growth factor of 1.05. Grids consisted of about 12,000. Grid independent analysis was undertaken to determine the grid sizes by examining the pressure drop of the modules via a hydrodynamic simulation.

The laminar model was chosen to describe the feed and permeate flows in the channels. The second-order upwind scheme was chosen to discrete the equations. The SIMPLE algorithm was used to solve the equations. The residuals of all the variables were set to 10^−6^.

The feed and permeate flow were governed by continuity equation, Navier-Stokes equations and energy equation,

(17) ∇ u=0

(18) $$\rho \left[\frac{\partial u}{\partial t}+\left(u\cdot \nabla \right)u\right]=-\nabla p+\nabla \cdot \sigma ,\sigma =\mu \left[\nabla u+{\left(\nabla u\right)}^{T}\right]$$

(19) $$\rho c_{p}\left[\frac{\partial T}{\partial t}+\left(u\cdot \nabla \right)T\right]=\nabla \cdot \left(k\nabla T\right).$$

where σ was the viscous stress tensor, ***u***, *p*, ρ were the velocity vector, pressure, density, respectively, μ was dynamic viscosity, *c*_p_ was the fluid heat capacity.

Mass transport was modeled using advection-diffusion equation,

(20) $$\frac{\partial c}{\partial t}+\left(u\cdot \nabla \right)c=\nabla \cdot \left(D\nabla c\right)$$

where *D* was the effective mass diffusivity.

Effective mass diffusivity *D* was obtained by [22],

(21) $$D\left(T\right)=17.872\times 10^{-14}\times \frac{\vartheta_{Na}\vartheta_{Cl}}{\vartheta_{Na}+\vartheta_{Cl}}T$$

(22) $$\vartheta_{Na}=50.11(1+0.02\left(T-298.15\right),\;\vartheta_{Cl}=76.35(1+0.02\left(T-298.15\right){,}_{\;}$$

where *ϑ_Na_* was the equivalent limiting ionic conductance of the sodium and *ϑ_Cl_* was the equivalent limiting ionic conductance of the chloride.

In order to simulate the transmembrane mass transfer process, it was necessary to load the mass source of water on the first layer grid near the permeate side and the feed side of the membrane. Besides, the transmembrane heat transfer process was achieved by loading the heat transfer source.

(23) $$S_{w}=\frac{N}{b},$$

where *b* was the height of the first grid. For the membrane distillation process, the mass source term on the liquid side was not only related to the feed side grid temperature, but also affected by the corresponding permeate side grid temperature, and it was achieved by the user-defined function (UDF).

The function of the UDF program was divided into three steps. First, the temperatures of cell center on both sides of the membrane wall was obtained by the UDF program, which were *T*_fm_, *T*_pm_, respectively. Then, the heat and mass transfer sources were calculated by UDF program through Equations (9)–(14). Finally, the UDF program was used to add the heat flux to membrane wall as boundary condition and mass transfer source as source terms.

### 3.2. Model Validation

**Figure 3:**
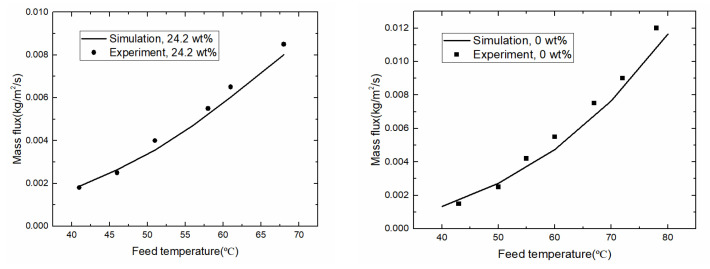
Model validation in different *T*_f_ (*c*_f_ = 24.2 wt.%, **left**; pure water, **right**).

The experimental data in the literature [18] were used to verify the model. In Figure 3, permeate fluxes were shown in different feed temperature for pure water and 24.2 wt.% NaCl solution, respectively. The simulation results agreed to the experiment data, which showed the results based on the current simulation model were reliable and the developed simulation methods were suitable for the following analysis.

## 4. Results and Discussion

In the current work, the influence of *T*_f_, *v*_f_, *v*_p_ on the temperature polarization in high-salt solution were simulated and analyzed. The effects of *T*_f_, *v*_f_, *v*_p_ and solution salinity on EE were studied. The optimal membrane thickness was discovered in different operation conditions.

### 4.1. Temperature Polarization

**Figure 4:**
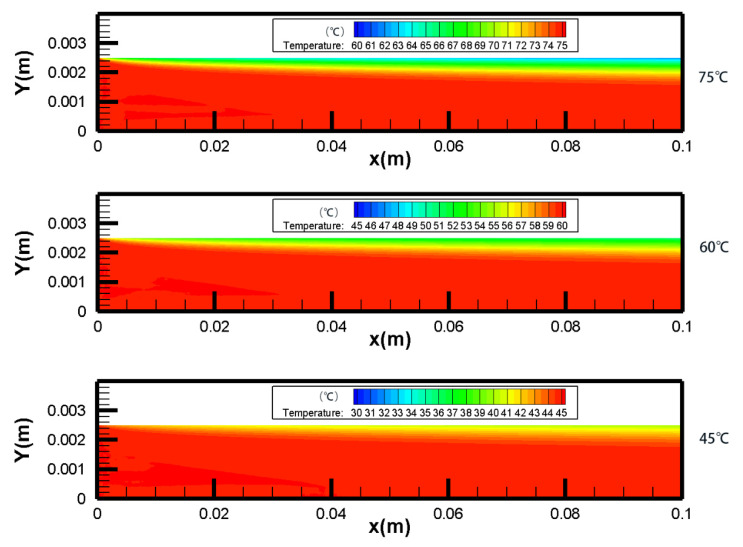
Contours of temperature distribution in different *T*_f_ (*v*_f_, *v*_p_ = 0.15 m/s, *T*_p_ = 25 °C, *c*_f_ = 24.2 wt.%).

**Figure 5:**
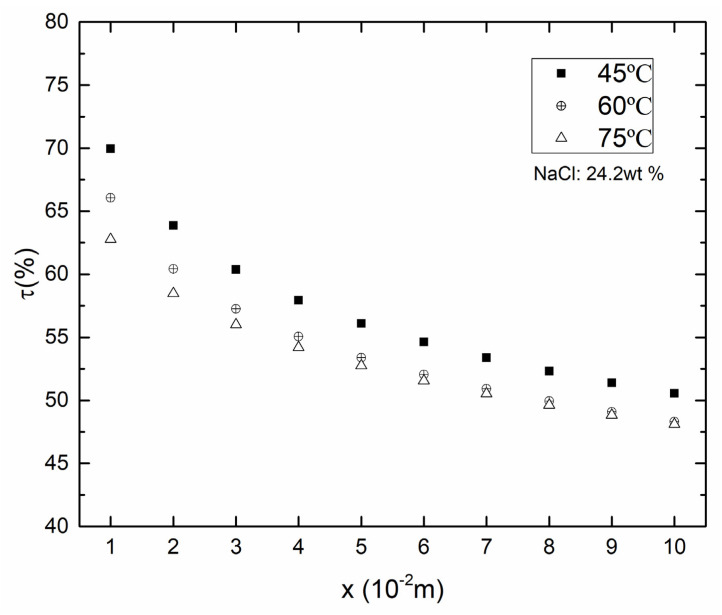
Temperature polarization coefficient in different *T*_f_ (*v*_f_, *v*_p_ = 0.15 m/s, *T*_p_ = 25 °C, *c*_f_ = 24.2 wt.%).

As shown in Figure 4 and Figure 5, there was a decrease in both TPC and *T*_f_ over the membrane surface along the x-axis in each *T*_f_. This was because that the temperature boundary layer became thicker along the x direction, and the temperature difference between the membrane surface and the bulk area became larger, which caused a decrease of TPC. With the *T*_f_ was increased from 45 to 75 °C, the TPC decreased correspondingly from 69.9% (x = 0.01 m) to 62.8% (x = 0.01 m). The variation of temperature difference between two sides of the membrane surface was smaller than that of the temperature difference between the bulk and membrane surface, which led to the decrease of TPC.

Ali’s [42] study showed the theoretical and experimental TPC as a function of Reynolds number (Re) in the range of 1000 to 5000. When the Re was below 1000, which was exactly the Re of current study, TPC was around 0.68, showing good coincidence with the current study.

**Figure 6:**
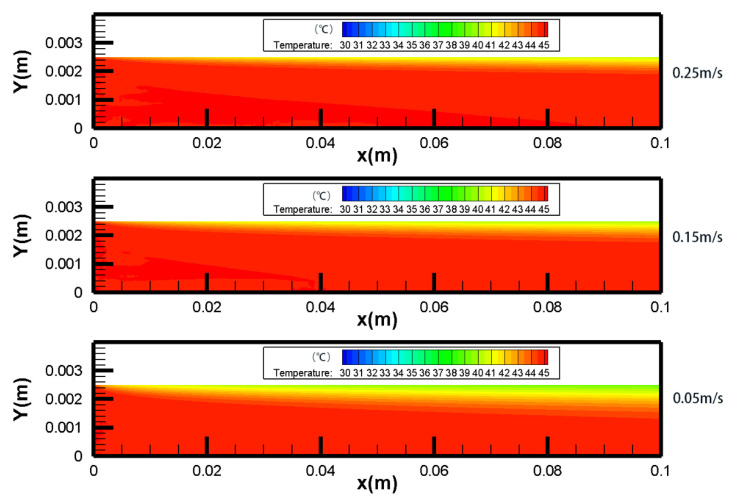
Contours of temperature distribution in different *v*_f_, *v*_p_ (*T*_f_ = 45 °C, *T*_p_ = 25 °C, *c*_f_ = 24.2 wt.%).

**Figure 7:**
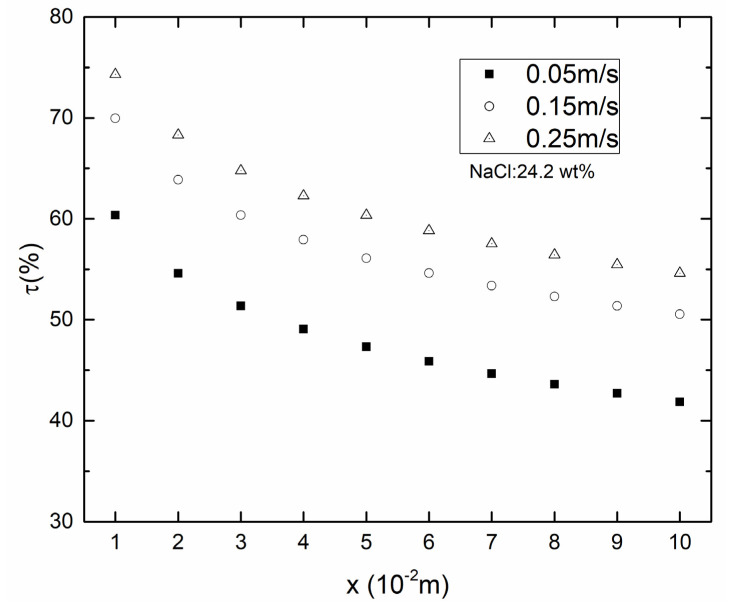
Temperature polarization coefficient in different *v*_f_, *v*_p_ (*T*_f_ = 45 °C, *T*_p_ = 25 °C, *c*_f_ = 24.2 wt.%).

As shown in Figure 6 and Figure 7, the temperature boundary layer became thinner with the increasing *v*_f_, *v*_p_. When the *v*_f_, *v*_p_ were 0.05 m/s, TPC decreased from 60.36% at inlet to 41.87% at outlet and the temperature difference between inlet and outlet at feed side was 5.86 °C. When *v*_f_, *v*_p_ were increased to 0.25 m/s, TPC decreased from 75.32% at inlet to 54.62% at outlet and the temperature difference between inlet and outlet at feed side was decreased to 4.56 °C. Results suggested that increasing the *v*_f_, *v*_p_ could reduce temperature polarization significantly.

### 4.2. Co- and Counter-Current Operations

In this section, in order to well investigate the temperature field and EE of co- and counter-current operations, the calculation domain of x direction was extended by 3 times. The influence of *v*_f_, *v*_p_, *T*_f_ and salinity on EE for co- and counter-current operation was evaluated in detail.

#### 4.2.1. Temperature Fields

**Figure 8:**
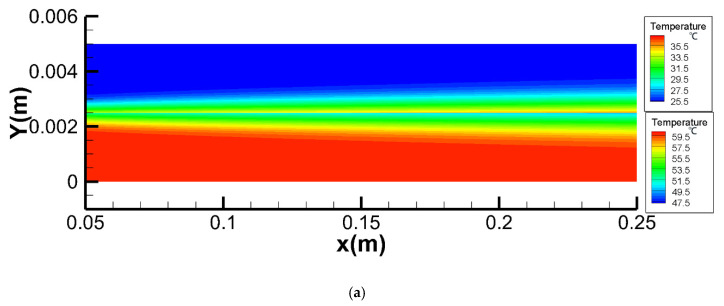

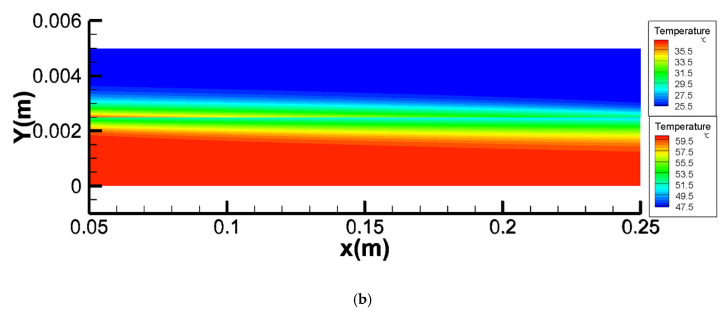
Temperature fields for co- and counter-current operations (different color scales were used in the permeate and feed channels, *T*_f_ = 60 °C, *T*_p_ = 25 °C, *v*_f_, *v*_p_ = 0.15 m/s, *c*_f_ = 24.2 wt.%). (**a**) co-current operation. (**b**) counter-current operation.

As shown in Figure 8, there was a decrease in *T*_f_ and an increase in *T*_p_ along the x-axis in co- and counter-current operations. In the studied cases, the feed side temperature difference between the inlet and outlet for co- and counter-current operations was 2.06 °C and 4.42 °C, respectively, which indicated that counter-current operation performed better than co-current. The temperature distribution of both feed and permeate sides along the x-axis was more homogeneous in counter-current operation, which resulted less thermal stress on the membrane. It was beneficial to prolong the working life of the membrane module. The temperature fields of count-current and co-current were well coincided with experimental data [43].

#### 4.2.2. Evaporation Efficiency for Co- and Counter-Current Operations

**Figure 9:**
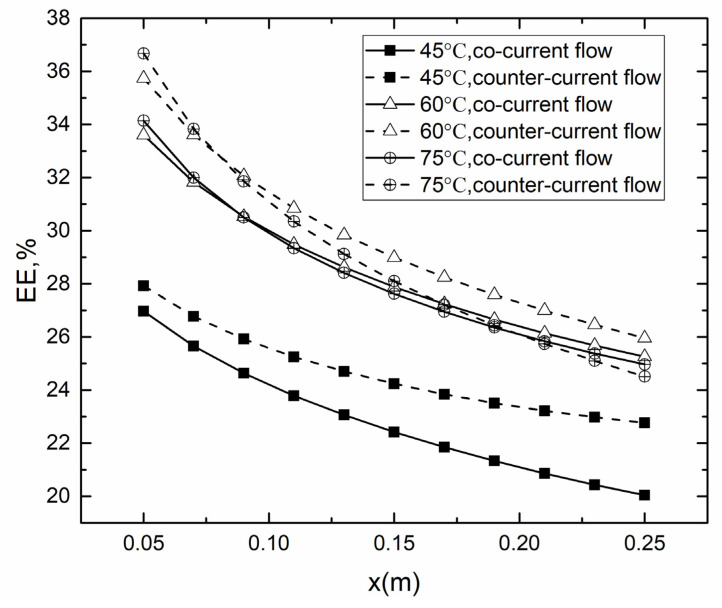
Evaporation efficiency (EE) for co- and counter-current operations in different *T*_f_. (*T*_p_ = 25 °C, *v*_f_, *v*_p_ = 0.15 m/s, *c*_f_ = 24.2 wt.%).

As shown in Figure 9, the EE was increased with the increase of *T*_f_ in all cases. It could be concluded that temperature difference was the main influencing factor for EE. Besides, there was a decrease of EE over the membrane surface along the x-axis due to the decrease of temperature. It was essential to control the length of membrane module to avoid the effect. Comparing the co- and counter-current operations in different *T*_f_, it was found that the counter-current operation performed better than co-current at upstream at all the studied temperatures. The priority of counter- to co-current operation at 45 °C was more evident than at 75 °C. This phenomenon could be attributed to several factors. In general, the temperature difference between feed and permeate membrane surface in counter-current operation was larger than co-current operation. The higher temperature difference lead to higher EE. According to Lou’s study [22], the concentration polarization of counter-current was less than that of co-current operation in the upstream and larger than that of co-current operation in the downstream.

**Figure 10:**
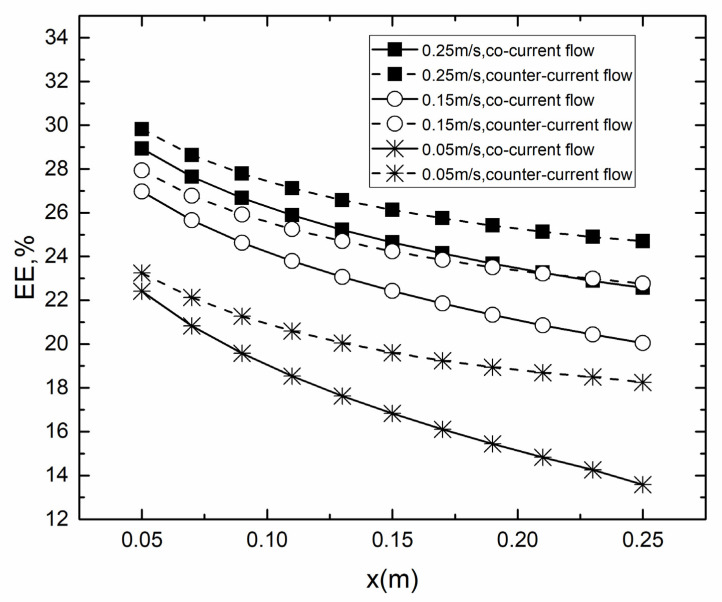
EE for co- and counter-current operations in different *v*_f_, *v*_p_. (*T*_f_ = 45 °C, *T*_p_ = 25 °C, *c*_f_ = 24.2 wt.%).

As shown in Figure 10, the decrease in EE with x-axis occurred in all cases due to the reduced temperature difference across the membrane and increased *c*_f_. The EE for counter-current operation performed better than that for co-current operation in the same *v*_f_, *v*_p_. With the *v*_f_, *v*_p_ increasing, the EE of both co- and counter-current increased. The difference of EE between counter- and co-current was decreasing along x-axis. When *v*_f_ and *v*_p_ were both 0.05 m/s, the EE for counter-current operation decreased slowly along the x-axis compared to co-current operation. This was due to the fact that the temperature different between the feed and permeate surface along x-axis was almost constant for counter-current operation and gradually decreased for co-current operation.

**Figure 11:**
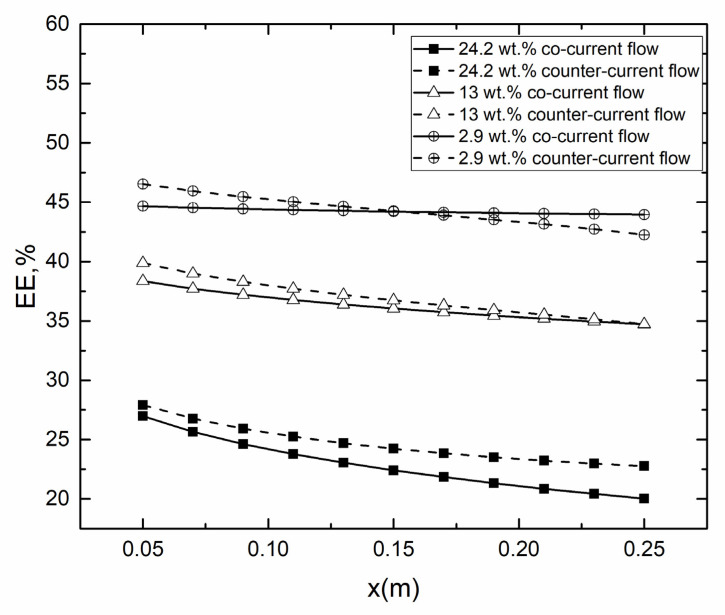
EE for co- and counter-current operations in different *c*_f_ (*T*_f_ = 45 °C, *T*_p_ = 25 °C, *v*_f_, *v*_p_ = 0.15 m/s).

As shown in Figure 11, it was found that the EE was generally low in high-salt solution due to the high concentration resulting in small permeate flux. It was worth noting that the EE for co-current operation started to higher than counter-current when the concentration was 2.9 wt.% at x = 0.15, which suggested that, in low concentrations, the impact of concentration polarization on EE was larger than temperature difference.

### 4.3. Optimal Membrane Thickness

Theoretically, two competitive effects are associated with membrane thickness. The thin membrane gives low mass transfer resistance and thus provide high transmembrane water flux at fixed operation condition. However, as the membrane thickness decreases, conductive heat loss through the membrane increases, leading to a low temperature gradient across the membrane and resulting low flux. Therefore, there must be an optimal thickness balancing the mass transfer resistance and conductive heat losses, and this thickness depends on operating conditions and other membrane characteristics.

Membrane thickness is an important property of membrane module but seldom studied [35,44,45,46]. Martinez [46] established a resistance-in-series model which was quite suitable to express the change of membrane thickness due to the import of transport resistance. Swaminathan [44] coupled the membrane thickness with the module size together and got the cost-optimal membrane thickness. Wu figured out the optimal membrane thickness both by analytical model and by experiment. The results indicated that the optimal thickness was much thicker than that could be made in experiment. Eykens [35] made a systematic research on membrane thickness. The influence of membrane thickness on DCMD at various salinities was investigated in detail. However, there were no studies involving in comparing of different membranes on membrane thickness and permeate flux.

The current study investigated the influence of membrane pore size, conductivity, tortuosity and porosity through comparing the different membrane materials, i.e., TF450 and HVHP, had the same pore size while the membrane conductivity, tortuosity and porosity were different, GVHP and HVHP had the same membrane conductivity, tortuosity and porosity while the pore size was different.

**Figure 12:**
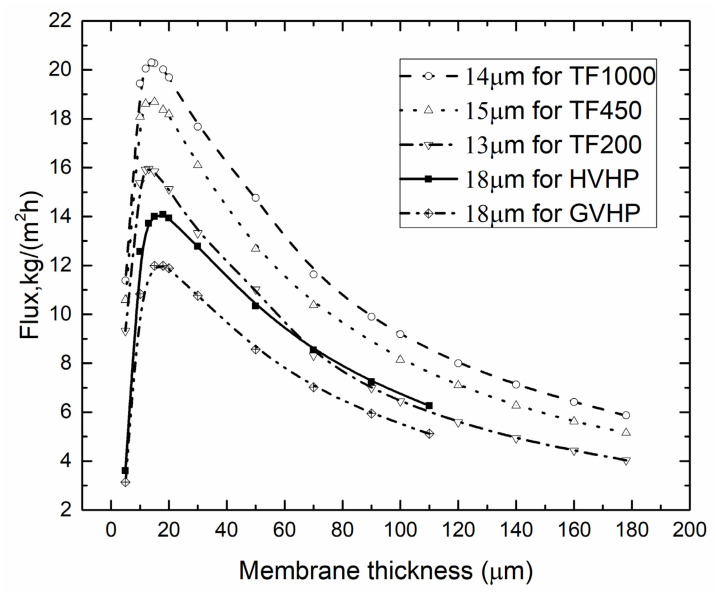
Optimal membrane thickness for different membrane materials (*T*_f_ = 45 °C, *T*_p_ = 25 °C, *v*_f_, *v*_p_ = 0.15 m/s, *c*_f_ = 24.2 wt.%).

Figure 12 showed the flux as function of membrane thickness for different membrane materials. There existed a threshold of permeate flux. By decreasing the membrane thickness, the permeate flux could be improved until reached this threshold. In Wu’s work [45], they found that the flux decreases with the decrease of membrane thickness through experiment, which was well reproduced in our simulations. The optimal membrane thickness and the largest permeate flux varied with different membrane materials. When the membrane thickness was kept as 15 μm, the permeate flux increased with the increasing of pore size for TF1000, TF450 and TF200, that was 20.30, 18.68, and 15.948 kg/(m^2^∙h), respectively. It indicated that the pore size had great impact on the permeate flux. The effect of thermal conductivity and porosity could be analyzed through TF450 and HVHP which had the same pore size, corresponding to permeate flux of 20.30 kg/(m^2^∙h) and 13.8 kg/(m^2^∙h). The thermal conductivity was 0.031 and 0.041 W∙m^−1^∙K^−1^ and the porosity was 0.8 and 0.75, respectively. Membrane material which had lower thermal conductivity and larger porosity showed higher permeate flux and thicker optimal membrane thickness. Lower thermal conductivity was helpful to reduce the heat loss, and larger porosity was beneficial to improving permeate flux. However, the optimal membrane thickness at 24.2 wt% was quite different with literature [30] (18 μm in the current work for GVHP and 49 μm in the literature for PP). The reason is that the tortuosity in our study is larger—the larger tortuosity means a thinner optimal membrane thickness. The membrane thermal conductivity is lower, the lower membrane conductivity also corresponds to a thinner optimal membrane thickness.

#### 4.3.1. Optimal Membrane Thickness for Different T_f_

**Figure 13:**
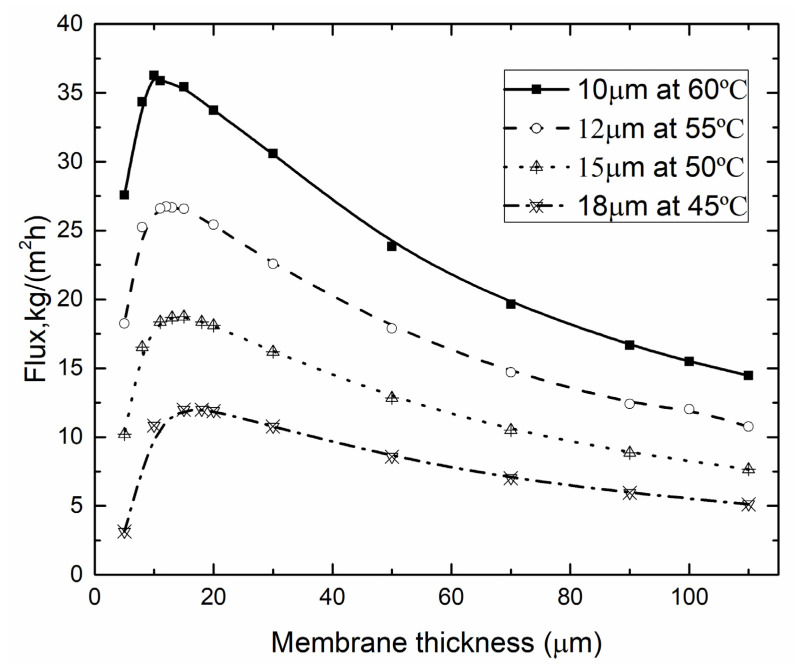
Optimal membrane thickness for different *T*_f_ (*T*_p_ = 25 °C, *v*_f_, *v*_p_ = 0.15 m/s, *c*_f_ = 24.2 wt.%, GVHP).

Figure 13 demonstrated the impact of *T*_f_ on the permeate flux and the membrane thickness for GVHP. Increase of permeate flux was observed when *T*_f_ increased from 45 to 60 °C. It was clearly seen that the higher *T*_f_ corresponded to the thinner membrane thickness. When *T*_f_ was 45 °C, the optimal membrane thickness was 18 μm. However, when *T*_f_ reached 60 °C, the optimal membrane thickness turned to 10 μm.

#### 4.3.2. Optimal Membrane Thickness for Different *v*_f_, *v*_p_

**Figure 14:**
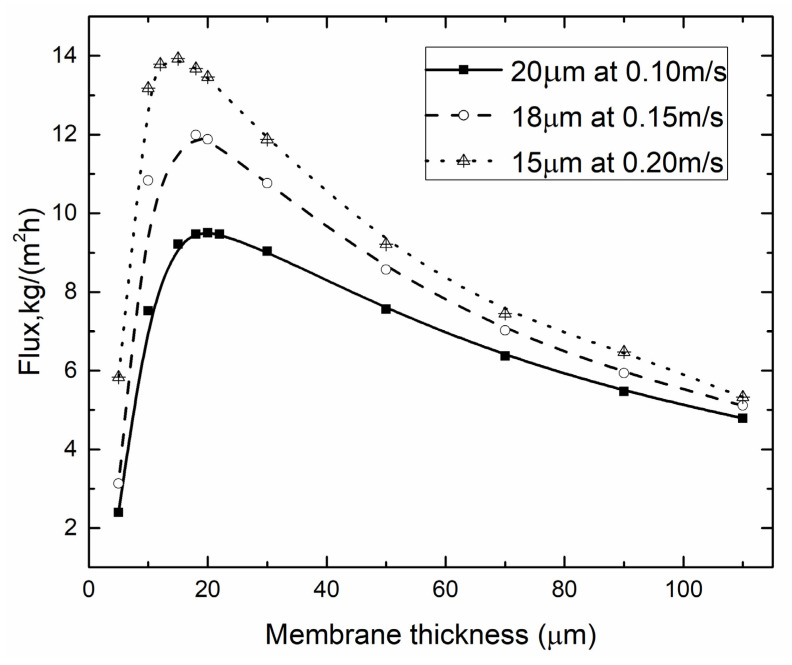
Optimal membrane thickness for different *v*_f_, *v*_p_ (*T*_f_ = 45 °C, *T*_p_ = 25 °C, *c*_f_ = 24.2 wt.%, GVHP).

As shown in Figure 14, the permeate flux was improved with the increasing of *v*_f_, *v*_p_. The optimal membrane thickness differed with different *v*_f_, *v*_p_. When *v*_f_, *v*_p_ was 0.1 m/s, the optimal membrane thickness was 20 μm, corresponding to permeate flux at 9.51 kg/(m^2^∙h), while these data became 15 μm corresponding to 13.93 kg/(m^2^∙h) when *v*_f_, *v*_p_ reached 0.2 m/s. The *v*_f_, *v*_p_ affected the optimal membrane thickness to a certain extent, but its impact was small.

The developed model could contribute to industrial application of MD, especially for supporting MD module designs in terms of optimization on membrane material and structure, working conditions or operating mode. In the current work, when the pore size varied from 0.2–1 μm, the Knudsen diffusion mechanism should be mainly considered for flux prediction, since the Knudsen diffusion coefficient is 50 times higher than that of molecular diffusion for the average pore size of 0.2 μm, and 10 times higher for the average pore size of 1 μm, when the membrane porosity is fixed. In the industries such as power plants, where a large amount of heat supply is present, the feed temperature should be properly increased to promote flux. In the industries such as for chemical synthesis with a low enthalpy of reaction, the selection of feed and permeate velocities, or pressure loss should be mainly considered for obtaining higher evaporation efficiency, where the model can be used for optimization by applying the conditions of the specific industries. Usually, counter-current operation performs better than co-current operation in terms of evaporation efficiency. Counter-current operation mode should be considered as priority. However, when the placement of MD module is geometrically limited and the aspect ratio of the membrane is high, counter-current mode is not necessarily better, the selection of co-current or counter-current mode can be determined by using simulation with the current model.

## 5. Conclusions

The simulation model coupling heat and mass transfer at the membrane surface through a user-defined function program was established. The optimal thickness for typical commercialized PVDF and PTFE membranes was studied, and it was found to be decreased with the increase of *v*_f_, *v*_p_ or *T*_f_. The TPC increased with the decrease of *T*_f_ or the increase of *v*_f_, *v*_p_. The counter-current operation mode performed better than co-current operation in most cases except for the condition where the *c*_f_ was low and the module length was long enough. The difference of EE between co- and counter-current was due to the combined effect of temperature difference and concentration polarization. In addition, membranes with lower thermal conductivity and larger porosity were prone to get higher permeate flux and thinner optimal membrane thickness. The developed model could also provide guidance for MD module designs.

## Figures and Tables

**Table 1:** Properties of the selected membranes.

Membrane Type	Producer	Material	*dp* (μm)	δ (μm)	*k_m_* (W∙m^−1^∙K^−1^)	ε	λ	Ref.
GVHP	Millipore	PVDF	0.22	120	0.041	0.75	2	[18]
HVHP	Millipore	PVDF	0.45	110	0.041	0.75	2	[18]
TF200	Gelman	PTFE	0.20	178	0.031	0.80	1.8	[33]
TF450	Gelman	PTFE	0.45	178	0.031	0.80	1.8	[33]
TF1000	Gelman	PTFE	1.00	178	0.031	0.80	1.8	[33]

## Abbreviations

**Nomenclature**	
*Q*	Heat flux, W∙m^−2^
*h*	Convective heat transfer coefficient, W∙m^−2^∙K^−1^
*T*	Temperature, K
*v*	Velocity, m∙s^−1^
*d_m_*	Hydraulic radius, m
Δ*H*	Latent heat of water, J∙kg^−1^
*N*	Mass flow across membrane, kg∙m^−2^∙s^−1^
*k*	Thermal conductivity, W∙m^−1^∙K^−1^
*k_m_*	Membrane thermal conductivity, W∙m^−1^∙K^−1^
*k_g_*	Thermal conductivity of air, W∙m^−1^∙K^−1^
*k_s_*	Thermal conductivity of membrane materials, W∙m^−1^∙K^−1^
D_w-a_	Diffusivity of water vapor-air mixture, m^2^∙s^−2^
D_kn_	Knudsen diffusion coefficient, m^2^∙s^−1^
P	Vapor pressure, Pa
*P_T_*	Total pressure, Pa
R	Gas constant (8.314 J∙mol^−1^∙K^−2^)
*x_s_*	Molar fraction of solute
*m_w_*	Molecular weight of water
b	Height of first grid, m
S_w_	Source, kg∙m^−^^3^∙s^−^^1^
**u**	Velocity vector, m∙s^−1^
*p*	Pressure, Pa
*x,y*	Cartesian coordinates, m
*c_p_*	Specific heat capacity, J∙kg^−1^∙K^−1^
*D*	Mass diffusivity, m^2^∙s^−1^
*c*	concentration, g∙L^−1^
*EE*	Evaporation Efficiency, %
*CPC*	Concentration polarization coefficient, %
*t*	Time, s
**Greek Symbols**	
*δ*	Membrane thickness, m
*ε*	Membrane porosity
*λ*	Membrane tortuosity
*γ_w_*	Activity coefficient of water
ρ	Density, kg∙m^−3^
σ	Viscous stress tensor
μ	Dynamic viscosity, Pa∙s
*ϑ_Na_*	The equivalent limiting ionic conductance of the sodium
*ϑ_Cl_*	The equivalent limiting ionic conductance of the chloride
**Subscripts**	
f	The bulk of the feed
fm	Membrane surface of the feed
p	The bulk of the permeate
pm	Membrane surface of the permeate
m	Membrane
N	Flux
C	Conduction
**Abbreviations**	
DCMD	Direct contact membrane distillation
FGD	Flue gas desulfurization
MD	Membrane Distillation
CFD	Computational fluid dynamics
CPC	Concentration polarization coefficient
TPC	Temperature polarization coefficient
UDF	User-defined function
EE	Evaporation efficiency
